# The Expression and Localisation of G-Protein-Coupled Inwardly Rectifying Potassium (GIRK) Channels Is Differentially Altered in the Hippocampus of Two Mouse Models of Alzheimer’s Disease

**DOI:** 10.3390/ijms222011106

**Published:** 2021-10-14

**Authors:** Rocío Alfaro-Ruiz, Alejandro Martín-Belmonte, Carolina Aguado, Félix Hernández, Ana Esther Moreno-Martínez, Jesús Ávila, Rafael Luján

**Affiliations:** 1 Synaptic Structure Laboratory, Instituto de Investigación en Discapacidades Neurológicas (IDINE), Departamento de Ciencias Médicas, Facultad de Medicina, Universidad Castilla-La Mancha, Campus Biosanitario, C/Almansa 14, 02008 Albacete, Spain; Rocio.Alfaro@uclm.es (R.A.-R.); Alejandro.Martin@uclm.es (A.M.-B.); Carolina.Aguado@uclm.es (C.A.); AnaEsther.Moreno@uclm.es (A.E.M.-M.); 2 Centro de Investigación Biomédica en Red Sobre Enfermedades Neurodegenerativas, ISCIII, 28049 Madrid, Spain; fhernandez@cbm.csic.es (F.H.); javila@cbm.csic.es (J.Á.); 3 Centro de Biología Molecular Severo Ochoa, CSIC-UAM, 28049 Madrid, Spain

**Keywords:** Alzheimer´s disease, hippocampus, GIRK channels, immunohistochemistry, electron microscopy, histoblot, P301S, APP/PS1, AD mouse model

## Abstract

G protein-gated inwardly rectifying K^+^ (GIRK) channels are the main targets controlling excitability and synaptic plasticity on hippocampal neurons. Consequently, dysfunction of GIRK-mediated signalling has been implicated in the pathophysiology of Alzheimer´s disease (AD). Here, we provide a quantitative description on the expression and localisation patterns of GIRK2 in two transgenic mice models of AD (P301S and APP/PS1 mice), combining histoblots and immunoelectron microscopic approaches. The histoblot technique revealed differences in the expression of GIRK2 in the two transgenic mice models. The expression of GIRK2 was significantly reduced in the hippocampus of P301S mice in a laminar-specific manner at 10 months of age but was unaltered in APP/PS1 mice at 12 months compared to age-matched wild type mice. Ultrastructural approaches using the pre-embedding immunogold technique, demonstrated that the subcellular localisation of GIRK2 was significantly reduced along the neuronal surface of CA1 pyramidal cells, but increased in its frequency at cytoplasmic sites, in both P301S and APP/PS1 mice. We also found a decrease in plasma membrane GIRK2 channels in axon terminals contacting dendritic spines of CA1 pyramidal cells in P301S and APP/PS1 mice. These data demonstrate for the first time a redistribution of GIRK channels from the plasma membrane to intracellular sites in different compartments of CA1 pyramidal cells. Altogether, the pre- and post-synaptic reduction of GIRK2 channels suggest that GIRK-mediated alteration of the excitability in pyramidal cells could contribute to the cognitive dysfunctions as described in the two AD animal models.

## 1. Introduction

G protein-gated inwardly rectifying K^+^ (GIRK) channels are a family of ion channels functionally coupled to PTX-sensitive G protein-coupled receptors (GPCRs), which generate slow inhibitory postsynaptic potentials [1,2]. Therefore, major roles of GIRK channels in the CNS are to mediate inhibitory responses by hyperpolarising neuronal membranes and decreasing neuronal excitability [2,3]. As downstream effectors of many GPCRs that use the G_i/o_ family of G proteins are known to be involved in >many physiological and pathological processes [4,5,6,7], disruption of the signalling mediated through GIRK channels has been implicated in the aetiology of neurological diseases, including Alzheimer’s disease (AD) [2,8]. Consequently, GIRK channels are now postulated as novel therapeutic targets for the development of new pharmacotherapies [9,10,11,12].

The mammalian GIRK family includes four subunits (GIRK1, GIRK2, GIRK3, and GIRK4) encoded by *KCNJ3*, *KCNJ6*, *KCNJ9*, and *KCNJ5* genes, respectively [1,13]. GIRK1, GIRK2, and GIRK3 are widely expressed in the CNS, whereas the expression of GIRK4 is generally lacking in the brain, with a few exceptions [14,15]. These subunits combine to form homomeric and heteromeric channels [16], although the molecular diversity of neuronal GIRK channels resulting from their combination is limited. Thus, the prototypical functional GIRK channel in the brain consists primarily of GIRK1 and GIRK2, as the genetic ablation of either of these subunits reduces the size of somato-dendritic GIRK currents in many neuron types [3,17]. Additionally, GIRK1 and GIRK2 can be co-immunoprecipitated, showed the same subcellular localisation patterns, and in regions where these subunits are co-expressed, the disruption of GIRK2 significantly reduces the expression of GIRK1 [10,18].

GIRK channels are highly expressed in brain regions associated with learning and memory, including the hippocampus [3,18] and are involved in synaptic plasticity [19], indicating a crucial role in cognitive function. The hippocampus is one of the brain regions firstly affected in AD, a disease characterised by the presence of senile plaques and neurofibrillary tangles (NFT), containing amyloid-β (Aβ) peptide and phospho-tau respectively, as well as synapse loss [20]. Using experimental models that reproduce some of these major neuropathological hallmarks in AD, previous studies have reported that Aβ induces a reduction in gene expression of hippocampal GIRK channel subunits and decreases GIRK conductance in pyramidal cells [21,22]. However, it remains unclear the role played by tau in GIRK channel expression and localisation.

Information regarding how GIRK channels reorganise at the surface of hippocampal neurons in AD and the neuronal pathways that are primarily affected is essential to understand the pathogenesis of the disease. For this purpose, we aim in this study to elucidate the protein levels and subcellular localisation of the GIRK2 subunit in the hippocampus of the P301S and APP/PS1 mouse models of tau pathology and Aβ pathology, respectively. Here we provide convincing evidence for a significant reduction in the expression of GIRK2 in the hippocampus of P301S mice, but not in APP/PS1 mice, and a loss of pre- and postsynaptic GIRK2 channels from the plasma membrane and accumulation at intracellular sites in CA1 pyramidal cells in the two transgenic mice.

## 2. Results

### 2.1. Brain Expression of GIRK2 Channels in P301S Mice

**Figure 1:**
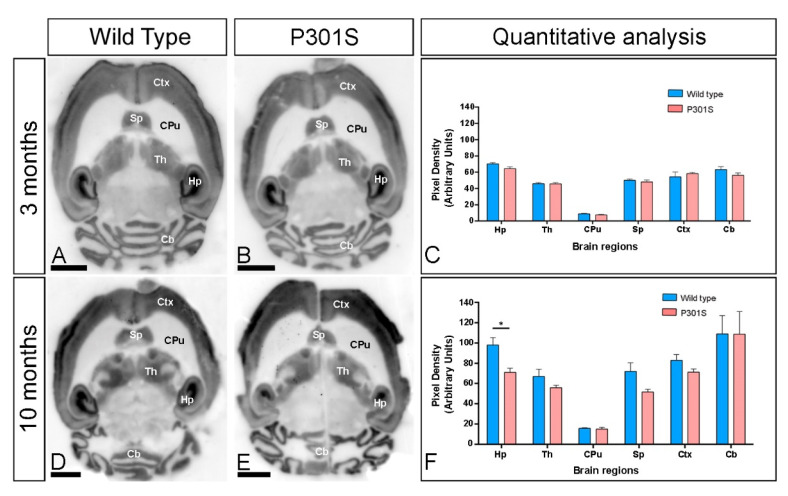
Regional expression of GIRK2 in the brain of P301S mice. (**A**–**F**) The expression of the GIRK2 protein was visualised in histoblots of horizontal brain sections at 3 and 10 months of age in wild type and P301S mice using an affinity-purified anti-GIRK2 antibody. GIRK2 exhibited broad distributions in the brain and region-specific differences were determined by densitometric analysis of the scanned histoblots (panels **C**,**F**). Strong GIRK2 staining was observed in the hippocampus (Hp), neocortex (Ctx), cerebellum (Cb), septum (Sp), and thalamus (Th), with moderate staining in midbrain nuclei and faint in the caudate putamen (CPu). Densitometric analysis showed no differences in GIRK2 expression in the brain of P301S mice at 3 months but showed a significant decrease in the hippocampus at 10 months of age (Multiple *t*-tests and Holm-Sidack method, * *p* < 0.05). Error bars indicate SEM. Scale bars: 0.2 cm.

We first determined the GIRK2 expression in the brain of P301S mice and age-matched wild type mice at 3 and 10 months using a GIRK2 subunit-specific antibody in conventional histoblotting [23]. In wild type mice, GIRK2 immunolabelling was widely distributed in the brain at the two ages studied, with strong labelling in the hippocampus, neocortex, cerebellum, septum, and thalamus (Figure 1A,C,D,F). Moderate labelling was observed in midbrain nuclei, including the inferior and superior colliculus, and brainstem nuclei (Figure 1A,D). Faint labelling was observed in basal ganglia nuclei such as the caudate putamen (Figure 1A,C,D,F). Qualitatively, this brain expression pattern was similar in the brain of P301S mice (Figure 1B,E). Quantitatively, analyses carried out to compare the protein expression levels revealed that this expression pattern of the wild type was unchanged in the brain of P301S mice at 3 months (Figure 1C) but showed a significant decrease in labelling in the hippocampus at 10 months of age (Figure 1F). 

### 2.2. Laminar Expression of GIRK2 Channels in the Hippocampus of P301S Mice

**Figure 2:**
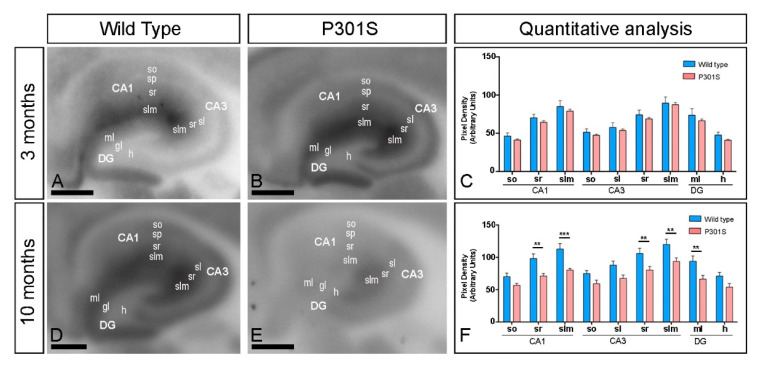
Hippocampal expression of GIRK2 in the hippocampus of P301S mice. (**A**–**F**) The expression of the GIRK2 protein was visualised in histoblots of horizontal brain sections at 3 and 10 months of age in wild type and P301S mice using an affinity-purified anti-GIRK2 antibody. GIRK2 expression in different hippocampal subfields and dendritic layers was determined by densitometric analysis of the scanned histoblots (panels C and F). GIRK2 expression in the CA1 region was strong in the *stratum lacunosum–moleculare* (slm) and distal half of the stratum *radiatum* (sr) and moderate in the proximal half of the stratum *radiatum* and the *stratum oriens* (so). In the CA3 region, expression for GIRK2 was strong in the *strata lucidum* (sl), *radiatum* (sr), and *lacunosum–moleculare* (slm), and moderate in the *stratum oriens* (so). In the DG, GIRK2 expression was strong in the molecular layer (ml) and weak in the hilus (h). Densitometric analysis showed no differences in GIRK2 expression in between wild-type and P301S mice of 3 months, but significant reduction in some dendritic layers at 10 months (Multiple t-tests and Holm-Sidack method, ** *p* < 0.01; *** *p* < 0.001). Error bars indicate SEM. *Abbreviations*: CA1 region of the hippocampus; CA3, CA3 region of the hippocampus; DG, dentate gyrus; so, *stratum oriens*; sp, *stratum pyramidale*; sr, *stratum radiatum*; slm, *stratum lacunosum-moleculare*; ml, molecular layer; gl, granule cell layer; h, hilus. Scale bars: 0.05 cm.

We next focused on the hippocampus, the brain region showing the significant reduction of GIRK2 expression and explored its laminar expression pattern using the histoblot technique (Figure 2A–F). In wild-type mice, GIRK2 was expressed in all hippocampal subfields and dendritic layers at the two ages (Figure 2A,D). The expression was strongest in the *stratum lacunosum–moleculare* in the CA1 region, with the stratum *radiatum* showing a moderate intensity in the proximal half and a strong intensity in the distal half, and with the *stratum oriens* also showing moderate intensity (Figure 2A,D). In the CA3 region, expression for GIRK2 was strongest in the *strata lucidum*, *radiatum* and *lacunosum–moleculare*, compared with the more moderate expression in the *stratum oriens* (Figure 2A,D). In the dentate gyrus, expression was strong in the molecular layer but weak in the hilus (Figure 2A,D). This expression pattern was very similar in all subfields and dendritic layers analysed between wild-type and P301S mice of 3 months (Figure 2B), which was confirmed using quantitative analyses (Figure 2C). However, the hippocampal expression of GIRK2 showed decrease in labelling at 10 months of age in a laminar-specific manner (Figure 2E). Quantitative analyses demonstrated that the expression of GIRK2 was reduced in the *strata radiatum* and *lacunosum-moleculare* of the CA1 and the CA3 regions, and the molecular layer of the dentate gyrus of P301S mice compared to age-matched wild type mice (Figure 2F).

Previous studies showed that GIRK2 forms macromolecular complexes with GIRK1, and both share similar distribution patterns in the hippocampus [24,25]. Therefore, it is expected that GIRK1 undergo similar changes in P301S mice at 10 months of age. Our analysis using histoblots showed that GIRK1 expression was very similar in the brain to that as described for GIRK2, with a significant decrease in labelling in the hippocampus at 10 months of age (Supplementary Figure S1). In the hippocampus, similarly to GIRK2, GIRK1 showed the same laminar distribution and decrease in labelling at 10 months of age in a laminar-specific manner (Supplementary Figure S1).

### 2.3. Similar Brain and Hippocampal Expression of GIRK2 Channels in APP/PS1 Mice

**Figure 3:**
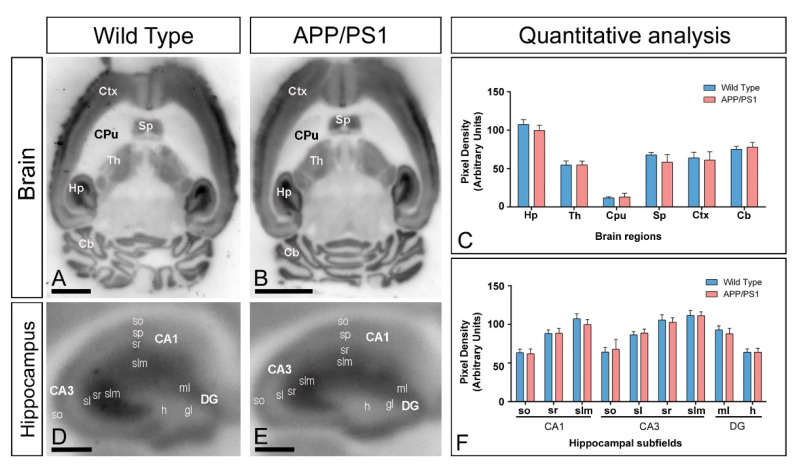
Regional expression of GIRK2 in the brain of APP/PS1 mice. (**A**–**C**) The regional brain expression was visualised in histoblots of horizontal brain sections at 12 months of age in wild type and APP/PS1 mice using an affinity-purified anti-GIRK2 antibody. The expression of GIRK2 revealed marked region-specific differences, with strongest immunoreactivity in the hippocampus (Hp), neocortex (Ctx), cerebellum (Cb), septum (Sp), and thalamus (Th) and weakest in the caudate putamen (CPu). Densitometric analysis showed no differences in GIRK2 expression in APP/PS1 mice compared to age-matched wild type controls. Error bars indicate SEM. (**D**–**F**) Hippocampal expression of GIRK2 in wild type and APP/PS1 mice visualised in histoblots of horizontal sections at 12 months of age. Expression for GIRK2 was strong in all dendritic layers of the CA1 and CA3 region and DG, with the *strata lacunosum–moleculare* (slm) and *radiatum* (sr) of the CA1 and CA3 regions and molecular layer (mL) of the DG showing the highest expression levels. A moderate expression was observed in the *stratum oriens* (so) of CA1 and CA3, and the *stratum lucidum* (sl) of CA3. The hilus (h) of the DG showed the lowest GIRK2 expression level in this region. Densitometric analysis showed no differences in GIRK2 expression in the hippocampal dendritic layers of APP/PS1 mice compared to age-matched wild type controls. Error bars indicate SEM. *Abbreviations*: CA1 region of the hippocampus; CA3, CA3 region of the hippocampus; DG, dentate gyrus; so, *stratum oriens*; sp, *stratum pyramidale*; sr, *stratum radiatum*; slm, *stratum lacunosum-moleculare*; ml, molecular layer; gl, granule cell layer; h, hilus. Scale bars: (**A**,**B**): 0.2 cm; (**D**,**E**): 0.05 cm.

To investigate whether the reduction in the GIRK2 expression observed in the tau model can be also detected in an Aβ mouse model, we used the histoblot technique to study GIRK2 in the brain of APP/PS1 mice at 12 months (Figure 3A–C). As described above, GIRK2 immunolabelling was strong in the hippocampus, neocortex, cerebellum, septum, and thalamus both in wild type (Figure 3A) and APP/PS1 mice (Figure 3B), showing no changes in regional expression patterns (Figure 3C). In the hippocampus, the GIRK2 immunolabelling in wild type and APP/PS1 mice exhibited similar laminar expression and distribution pattern as that described for P301S mice (Figure 3D,E). Our quantitative analysis confirmed that the expression levels of GIRK2 in all subfields and dendritic layers analysed was unchanged in APP/PS1 mice compare to age-matched wild type controls (Figure 3F).

### 2.4. Altered Postsynaptic Localisation of GIRK2 in the Hippocampus of Transgenic Mice

**Figure 4:**
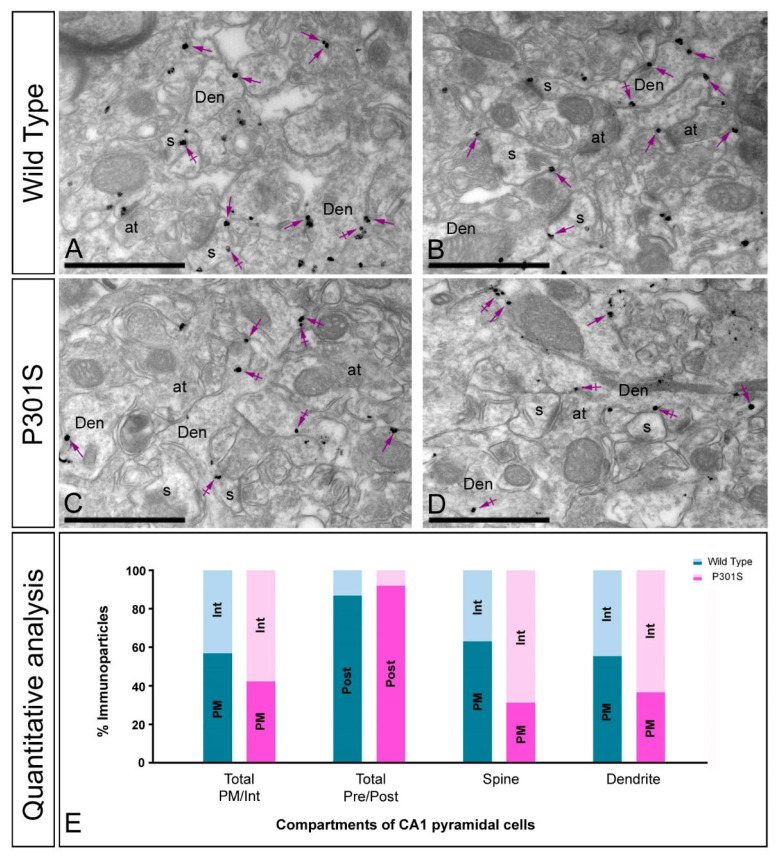
Changes in the postsynaptic localisation of GIRK2 in the hippocampus of P301S mice. Electron micrographs showing immunoparticles for GIRK2 in the *stratum radiatum* of the CA1 region at 10 months of age in wild type and P301S mice, as detected using a pre-embedding immunogold technique. (**A**–**D**) In wild type mice, immunoparticles for GIRK2 were mostly located at the extrasynaptic membrane (arrows) of dendritic spines (s) and shafts (Den) of CA1 pyramidal cells, as well as at intracellular sites (crossed arrows). In P301S mice, fewer immunoparticles for GIRK2 were detected along the extrasynaptic membrane (arrows) of dendritic spines (s) and shafts (Den) of pyramidal cells, with more GIRK2 immunoparticles being observed at intracellular sites (crossed arrows). (**E**) Quantitative analysis performed in different compartments of CA1 pyramidal cells showing that immunoparticles for GIRK2 were less frequently observed along the extrasynaptic plasma membrane of dendritic spines and shafts and more frequently at intracellular sites in P301S mice. Scale bars: (**A**–**D**): 1 μm.

**Figure 5:**
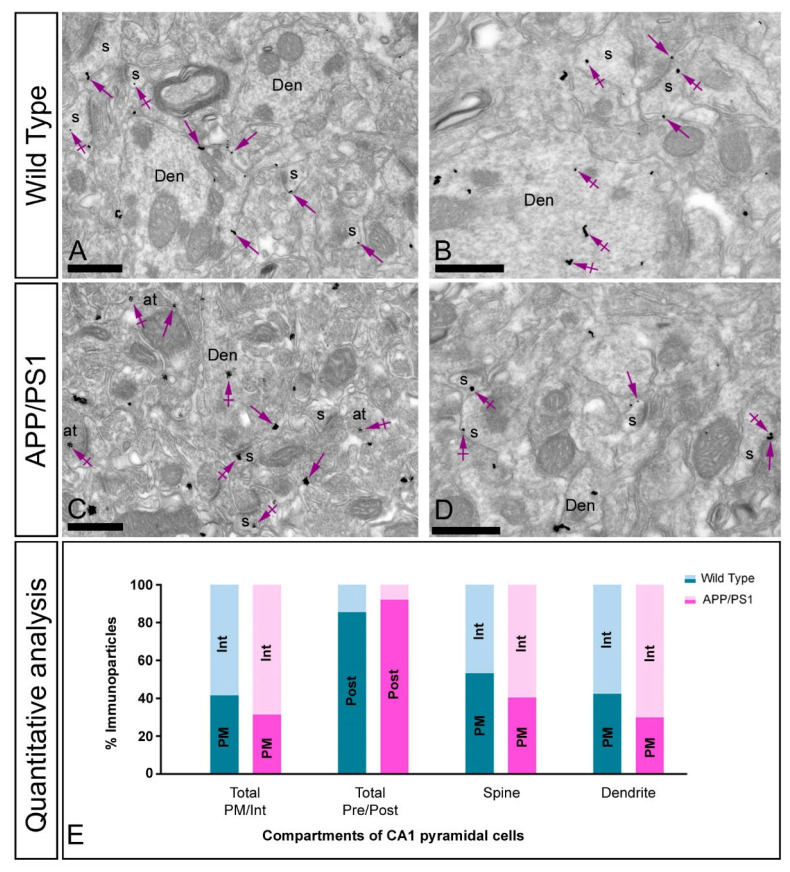
Changes in the postsynaptic localisation of GIRK2 in the hippocampus of APP/PS1 mice. Electron micrographs showing immunoparticles for GIRK2 in the *stratum radiatum* of the CA1 region at 12 months of age in wild type and APP/PS1 mice, as detected using a pre-embedding immunogold technique. (**A**–**D**) In wild type mice, GIRK2 immunoparticles were mostly located along the extrasynaptic plasma membrane (arrows) of dendritic spines (s) and shafts (Den) of CA1 pyramidal cells, and at intracellular sites (crossed arrows). In APP/PS1 mice, GIRK2 immunoparticles were less frequently observed along the extrasynaptic membrane (arrows) of dendritic spines (s) and shafts (Den), but more frequently detected at intracellular sites (crossed arrows). (**E**) Quantitative analysis of immunoparticles for GIRK2 in different compartments of CA1 pyramidal cells demonstrated the reduction of labelling along the extrasynaptic plasma membrane and the increase at intracellular sites in APP/PS1 mice. Scale bars: (**A**–**D**): 500 nm.

To investigate how the GIRK2 subunit is organised in different compartments of pyramidal cells in normal and pathological conditions, its subcellular localisation was explored in the CA1 region of hippocampal sections obtained from 10 months old wild-type and P301S mice (Figure 4) and from 12 months old wild-type and APP/PS1 mice (Figure 5). The analysis was carried out in the *stratum radiatum*, a subfield exhibiting prominent labelling for GIRK2.

Immunoreactivity for the GIRK2 subunit in wild type mice was primarily detected in postsynaptic elements, namely, on dendritic spines and shafts of CA1 pyramidal cells (Figure 4A,B and Figure 5A,B). Immunoparticles were localised at the extrasynaptic plasma membrane of dendritic spines in contact with axon terminals, likely deriving from Schaffer collaterals, and dendritic shafts, as well as at intracellular sites associated with the endoplasmic reticulum cisterna (Figure 4A,B and Figure 5A,B). In both P301S and APP/PS1 mice, immunoparticles for GIRK2 were observed in the same subcellular compartments as in wild type (Figure 4C,D and Figure 5C,D), but more frequently detected intracellularly, as demonstrated from the quantitative analyses (Figure 4E and Figure 5E). This analysis showed significant differences in frequency of plasma membrane-associated versus intracellular GIRK2 immunolabelling in CA1 pyramidal cells in both P301S mice (Plasma membrane: 57% in wild type, *n* = 501 particles, and 42% in P301S, *n* = 209 particles; Intracellular: 43% in wild type, *n* = 414 particles, and 58% in P301S, *n* = 284 particles) and APP/PS1 mice (Plasma membrane: 42% in wild type, *n* = 617 particles, and 32% in APP/PS1, *n* = 854 particles; Intracellular: 58% in wild type, *n* = 802 particles, and 68% in APP/PS1, *n* = 1878 particles) (Figure 4E and Figure 5E). These changes in subcellular localisation from the plasma membrane to intracellular sites were detected both in dendritic spines and dendritic shafts of CA1 pyramidal cells (Figure 4E and Figure 5E), thus demonstrating a redistribution of GIRK2 in hippocampal principal cells.

### 2.5. Altered Presynaptic Localisation of GIRK2 in the Hippocampus of Transgenic Mice

**Figure 6:**
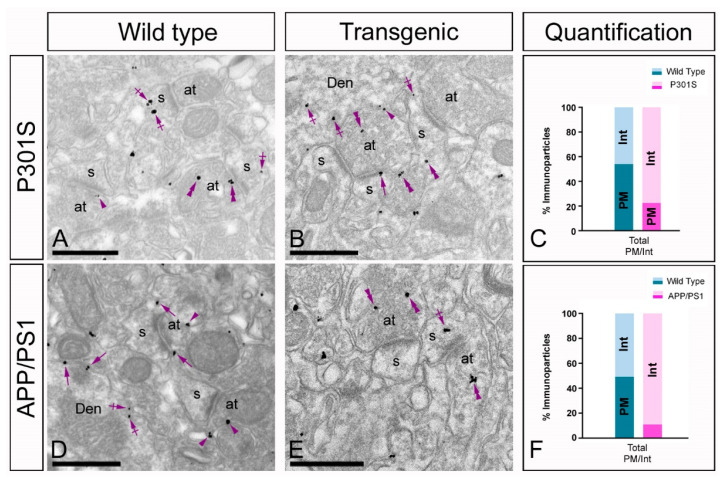
Changes in the presynaptic localisation of GIRK2 in the hippocampus of P301S and APP/PS1 mice. Electron micrographs illustrating presynaptic immunoparticles for GIRK2 in the stratum radiatum of the CA1 region in 10 months old in wild type and P301S mice at and 12 months old in wild type and APP/PS1 mice, as detected using a pre-embedding immunogold technique. (**A**–**F**) In wild type mice (panels **A**,**D**), immunoparticles for GIRK2 were found at presynaptic sites, where they localised at the extrasynaptic plasma membrane (arrows) of axon terminals (at) establishing asymmetrical synapses with spines (s), and at intracellular sites (crossed arrows). Both in P301S and APP/PS1 mice (panels **B**,**E**), immunoparticles for GIRK2 found at presynaptic sites in axon terminals were more frequently detected at intracellular sites. Arrows and crossed arrows indicate to GIRK2 immunoparticles in dendritic spines (s) and shafts (Den) at extrasynaptic and intracellular sites, respectively. Scale bars: (**A**,**B**,**D**,**E**): 500 nm.

To investigate whether the GIRK2 subunit undergoes changes at presynaptic sites in pathological conditions, its subcellular localisation was studied in the *stratum radiatum* of the CA1 region in P301S and APP/PS1 mice (Figure 6A–F). Immunoreactivity for GIRK2 was also detected in presynaptic elements, namely, on axon terminals establishing excitatory synapses with dendritic spines of CA1 pyramidal cells in both P301S and APP/PS1 mice (Figure 6A,B,D,F). Immunoparticles were localised to the active zone and to the extrasynaptic plasma membrane of the axon terminals (Figure 6A,D). In both P301S and APP/PS1 mice, most presynaptic immunoparticles for GIRK2 were observed at intracellular sites (Figure 6B,E). This change in the presynaptic localisation of GIRK2 was demonstrated using quantitative approaches (Figure 6C,F). The immunoparticles found at presynaptic sites showed that most (89% in P301S, *n* = 18; 77% in APP/PS1, *n* = 211) were found intracellularly in axon terminals (Figure 6C,F).

We also calculated the density of pre- and postsynaptic immunoparticles for GIRK2 in the sampling area of the *stratum radiatum* used for the above quantitative analyses. In a total sampling area of 500 µm^2^, the density of GIRK2 was 1.76 immunoparticles/µm^2^ in wild type and 1.11 immunoparticles/µm^2^ in P301S. This significant reduction in the density of GIRK2 is consistent with the reduction in expression described with histoblot. In APP/PS1 mice, the density of GIRK2 was not significantly different (1.82 immunoparticles/µm^2^ in wild type and 1.77 immunoparticles/µm^2^ in APP/PS1), consistent with the unaltered expression of GIRK2 protein observed using histoblot.

## 3. Discussion

GIRK channel activation in the hippocampus provides a major inhibitory pathway that is important in physiological and pathological states. Given that GIRK channels play a crucial role in cognitive function [26], their pathophysiological alterations have been demonstrated in AD [21,27,28,29], thus highlighting their potential therapeutic applicability [9,12]. In the present work, our goal was to investigate if the expression and subcellular localisation of the GIRK2 subunit undergoes changes in two transgenic models of AD. We have quantified the expression of GIRK2 channels within the hippocampus of two transgenic mice and determined their reduction in expression in P301S mice at 10 months but not in APP/PS1 mice at 12 months. At the ultrastructural level, our data provide the first detailed description of the altered localisation of GIRK2 channels in the hippocampus in the two transgenic mouse models, presenting quantitative evidence that a reduction in GIRK2 takes place both in postsynaptic and presynaptic compartments of CA1 pyramidal cells. The reported decrease of GIRK2 channels along the plasma membrane in both P301S and APP/PS1 mice may be one of the contributing factors to the synaptic dysfunctions and memory deficits in the two AD models.

### 3.1. Differential Expression Pattern of GIRK Channels in Transgenic Mice

Previous in situ hybridisation and immunohistochemical reports have shown that the GIRK2 subunit is the most abundant subunit of GIRK channels in the hippocampus [14,25,30,31,32], where it plays a crucial role in the assembly and surface localisation of functional GIRK channels [33]. Accordingly, we have shown by histoblot that GIRK2 was widely expressed in the hippocampus at ages of 3, 10 and 12 months and the labelling was particularly strong in dendritic layers, consistent with previous studies carried out in young adult mice [32].

Changes in protein expression have been reported to take place in AD pathology, but information on the signalling proteins and neuronal pathways affected is scarce. Given the limitations of investigating AD in human subjects, to understand the underlying molecular mechanisms of this disorder current studies mostly rely on animal models resembling all stages of disease progression. Pathological studies suggest that neuronal dysfunction caused by Aβ oligomers ultimately results in NFT formation and neuron death [34]. Furthermore, increasing evidence is linking Tau and Aβ in synaptic dysfunction [35,36]. Here, to determine the potential pathophysiological relevance of Tau and Aβ in neuronal GIRK channels, we first investigated whether Tau pathology and Aβ pathology could result in a change in the expression of GIRK2. One important finding is that the GIRK2 protein expression is reduced in the hippocampus of P301S mice. This alteration is taking place in a laminar-dependent manner, mainly affecting dendritic compartments related with the perforant pathway. Given that the AD pathologic increase of phospho-Tau and Aβ in the entorhinal cortex, which induce synaptic weakening in the hippocampus [35,37], laminar differences in the GIRK2 expression suggest a potential circuit specificity in the alteration, and related with entorhinal cortical pathology. Additionally, we also detected a strikingly similar reduction in the expression of the GIRK1 subunit in the hippocampus of P301S mice. This data, together with the dramatic reduction of GIRK1 labelling observed in the hippocampus of GIRK2 KO mice [25], is consistent with the idea that a significant fraction of hippocampal GIRK channels altered in AD are heteromultimers composed of GIRK1 and GIRK2. The relevance of GIRK3 to hippocampal GIRK channels in AD remains an interesting subject that deserves further investigations.

In the present study, a decrease in GIRK2 protein expression was not detected in the hippocampus of APP/PS1 mice, consistent with the unaltered mRNA and protein expression of GIRK1 Aβ (1–42)-infused rat model of AD [38]. However, previous studies using different models showed that Aβ induces a reduction in gene expression of hippocampal GIRK channel subunits and decreases GIRK conductance in pyramidal cells [21,22] and a reduction of protein expression in the human hippocampus of AD [27]. Whilst are data contrasts with other studies, this may be due to the fact that we used different models of AD, which recapitulate distinct features of the disorder. Moreover, we cannot rule out the possibility that these changes in expression are the result of the significant neuronal loss associated with P301S mice [39], which in APP/PS1 mice is only present adjacent to Aβ plaques [40].

### 3.2. Altered Postsynaptic Localisation of GIRK Channels in Transgenic Mice

Neuronal GIRK channels mediate the postsynaptic inhibitory effects of many neurotransmitters and drugs of abuse that target G_i/o_-coupled receptors [41]. Here, we provide direct evidence that GIRK2 is found predominantly at postsynaptic sites in dendritic compartments of CA1 pyramidal cell in the hippocampus, which is consistent with previous studies in the hippocampus, neocortex, substantia nigra and cerebellum [24,25,31,42]. Our ultrastructural data support the main postsynaptic role of GIRK channels as described in previous electrophysiological studies [3] and is consistent with functional data which shows that GIRK-mediated currents are larger in dendrites than in other compartments of hippocampal neurons [42].

Perhaps the most striking finding of the quantitative immunoelectron microscopic analyses undertaken in this study is the reduction in the frequency of immunoparticles for GIRK2 on the plasma membrane of CA1 pyramidal cells in the hippocampus of both P301S and APP/PS1 mice, accompanied by an increase of immunoparticles at intracellular sites. In comparing the subcellular localisation of GIRK2 in the two animal models of AD, we show that the GIRK channel subunit labels intracellular sites more frequently than the plasma membrane. Interestingly, recent studies reported strikingly similar subcellular changes for GABA_B_ receptors in the same compartments of CA1 pyramidal cells in APP/PS1 mice [27]. One of the best characterised effectors modulated by GABA_B_ receptors is the GIRK channel, whose activation is responsible for the slow inhibitory postsynaptic potentials IPSP in hippocampal pyramidal cells [3]. This functional association is facilitated by the formation of stable macromolecular complexes [25,43]. Altogether, our data suggest that Aβ pathology induce an impartment in the excitability of pyramidal cells mediated by GABA_B_-GIRK signalling. Based on the similar GIRK2 reductions at ultrastructural level, it is expected that Tau pathology induce similar alterations.

Whilst the two transgenic mouse models we used for this study, revealed at the qualitative level, a reduction of GIRK2 along the plasma membrane and accumulation at intracellular sites, it is important to note that immunoparticles for GIRK2 in the hippocampus of P301S were found at lower frequencies than in APP/PS1 mice. Such difference could be explained by the neuronal loss observed in the former mouse model [39] and is consistent with the reduction in expression found using histoblots. In APP/PS1 mice we did not detect any change in the total protein expression, consistent with the similar density of immunoparticles detected using immunoelectron microscopic techniques. However, Aβ pathology induced a change in the subcellular localisation of GIRK2 from plasma membrane to intracellular sites. Similar subcellular changes have been described for different receptors in the same compartments of CA1 pyramidal cells in APP/PS1 mice [27,44].

### 3.3. Altered Presynaptic Distribution of GIRK Channels in Transgenic Mice

In addition to its main postsynaptic distribution, a small but consistent presynaptic labelling for GIRK2 was also detected in glutamatergic axon terminals establishing synapses with CA1 neurons, consistent with previous observations in many brain regions [18]. Presynaptic inhibition in the hippocampus results primarily from the G-protein-dependent regulation of voltage-gated Ca^2+^ channel activity [45], but the location of GIRK channels on the extrasynaptic and synaptic plasma membrane of excitatory terminals suggests their involvement in the regulation of neurotransmitter release. Although electrophysiological studies did not support a role for presynaptic GIRK channels [3], using functional assays we have elucidated a role for the GIRK channel-mediated inhibition of glutamate release through GABA_B_ receptors at cerebrocortical and cerebellar nerve terminals [24,46].

Although most studies dealing with the implication of Aβ and tau in synaptic dysfunction have concentrated in postsynaptic receptors and dendritic mechanisms [36], increasing evidence suggest that deficits in presynaptic mechanisms are also present in AD [47,48]. A notable finding of our quantitative ultrastructural studies is the decrease of presynaptic GIRK2 in axon terminals from Schaffer collaterals in both P301S and APP/PS1 mice. Consistent with our results, evidence from immunoelectron microscopic studies in the APP/PS1 mouse model suggests that GABA_B_ receptors located at presynaptic sites are also reduced in the Schaffer collateral synapses [27]. Previous reports indicated that the increase in Aβ production is linked to reduced GABA_B_ receptor expression in the APP-/- mice [49] and that secreted APP binds to GABA_B_ receptors to supress synaptic vesicle release, thus modulating synaptic transmission and plasticity [50]. Given that GABA_B_ receptors are molecularly and functionally coupled with GIRK channels, mainly the GIRK1 and GIRK2 subunits, in the hippocampus [25,42,51], impairment of presynaptic mechanisms through dysfunction of GABA_B_-GIRK signalling is likely to greatly influence the activity of neural circuits and can potentially participate in the pathogenesis observed in both P301S and APP/PS1 mouse models of AD.

In summary, the work presented here supports the evidence that phospho-Tau and Aβ can modulate the expression and localisation of hippocampal GIRK2-containing channels in the AD pathogenesis. Our quantitative analyses demonstrate for the first time how the subcellular localisation of GIRK2 channels is altered both pre- and postsynaptically in P301S and APP/PS1 mice. The pre- and postsynaptic reduction of GIRK channel in pyramidal cells may represent the molecular and anatomical substrate of the altered cognitive function observed in the two mouse models of AD. These findings highlight the functional importance of this change in subcellular localisation because it implies a specific lack of functions for GIRK2 in the modulation of neuronal excitability in pathological conditions.

## 4. Material and Methods

### 4.1. Animals

We used transgenic mice P301S for the human tau gene and wild type control littermates. The P301S mouse model, obtained from Jackson laboratory (B6;C3-Tg(Prnp-MAPT*P301S)PS19Vle/J), carries a mutant (P301S) human MAPT gene encoding T34-tau isoform (1N4R) driven by the mouse prion-protein promoter (Prnp) on a B6C3H/F1 genetic background. For analysis, we selected animals of 3 months of age, characterised by no sign of pathology [39], and 10 months of age, characterised by widespread neurofibrillary tangles accumulation, impaired memory, spatial learning and LTP, impaired synaptic function and neuronal loss [39]. A total of 12 mice aged 3 months (*n* = 3 for WT, *n* = 3 for P301S for histoblotting; and *n* = 3 for WT, *n* = 3 for P301S for immunoelectron microscopy) and 12 mice aged 10 months (*n* = 3 for WT, *n* = 3 for P301S for histoblotting; and *n* = 3 for WT, *n* = 3 for P301S for immunoelectron microscopy) were analysed. All mice were housed at the “Centro de Biología Molecular Severo Ochoa” animal facility. Mice were housed four per cage with food and water available ad libitum and maintained in a temperature-controlled environment on a 12/12 h light–dark cycle with light onset at 07:00 h. Animal housing and maintenance protocols followed the guidelines of Council of Europe Convention ETS123, recently revised as indicated in Directive 86/609/EEC. Animal experiments were performed under protocols (P15/P16/P18/P22) approved by the Institutional Animal Care and Utilisation Committee (Comité de Ética de Experimentación Animal del CBM, CEEA-CBM, Madrid, Spain). 

In addition, male APP/PS1 mice (RRID:IMSR_MMRRC:034832) were obtained from the Jackson Laboratory (https://www.jax.org/strain/005864) and expressed Mo/Hu APP695s. we construct in conjunction with the exon-9-deleted variant of human presenilin 1 [Tg(APPswe,PSEN1dE9)85Dbo/Mmjax] [40,52]. The “control” wild type (WT) mice were age-matched littermates without the transgene. For analysis, we selected animals of 12 months of age, characterised by memory deficits with severe synapse loss and widespread Aβ deposition [53,54]. A total of 12 mice (*n* = 3 for WT, *n* = 3 for APP/PS1 for histoblotting; and *n* = 3 for WT, *n* = 3 for APP/PS1 for immunoelectron microscopy) were analysed. All mice were maintained at the Animal House Facility of the University of Castilla-La Mancha (Albacete, Spain) in cages of 2 or more mice, on a 12-h light/12-h dark cycle at 24 °C and received food and water ad libitum. Care and handling of animals prior to and during experimental procedures were in accordance with Spanish (RD 1201/2015) and European Union regulations (86/609/EC), and all protocols and methodologies were approved by the local Animal Care and Use Committee.

For histoblotting, animals were deeply anesthetised by intraperitoneal injection of ketamine/xylazine 1:1 (ketamine, 100 mg/kg; xylazine, 10 mg/kg), the hippocampus was dissected, frozen rapidly in liquid nitrogen and stored at −80 °C. For immunohistochemistry experiments at the electron microscopic level, using the pre-embedding immunogold technique, animals were firstly deeply anaesthetised by intraperitoneal injection of ketamine-xylazine 1:1 (ketamine, 100 mg/kg; xylazine, 10 mg/kg) and then transcardially perfused with ice-cold fixative containing 4% (*w*/*v*) paraformaldehyde with 0.05% (*v*/*v*) glurataldehyde in 0.1 M phosphate buffer (PB, pH 7.4) for 15 min. After perfusion, brains were removed from the skull and tissue blocks were washed thoroughly in 0.1 M PB. Coronal 60 μm thick sections were cut on a Vibratome (Leica V1000, Leica, Wetzlar, Germany).

### 4.2. Antibodies and Chemicals

We used a guinea pig anti-GIRK2 polyclonal antibody (GP-Af830; aa. 390–421 of mouse GIRK2A-1; RRID: AB_2571713; Frontier Institute Co., Hokkaido, Japan) and a rabbit anti-GIRK1 polyclonal antibody (Rb-Af530; aa. 469–501 of mouse GIRK1 C-terminal; RRID: AB_2571711; Frontier Institute Co., Japan). The preparation, purification, full characterisation, and specificity of these antibodies have been described previously [24,55]. The secondary antibodies used were as follows: alkaline phosphatase (AP)-goat anti-guinea pig IgG (H+L) (1:5000; Sigma-Aldrich, Sant Louis, MO, USA), and goat anti-guinea pig IgG coupled to 1.4 nm gold (1:100; Nanoprobes Inc., Stony Brook, NY, USA).

### 4.3. Histoblotting

The regional distribution of GIRK2 was analysed in P301S and APP/PS1 mouse brains using the histoblot technique [23]. Briefly, horizontal cryostat sections (10 µm) from mouse brains (wild type and transgenics) were apposed to nitrocellulose membranes moistened with 48 mM Tris-base, 39 mM glycine, 2% (*w*/*v*) sodium dodecyl sulphate and 20% (*v*/*v*) methanol for 15 min at room temperature (~20 °C). After blocking in 5% (*w*/*v*) non-fat dry milk in phosphate-buffered saline, nitrocellulose membranes were treated with DNase I (5 U/mL), washed and incubated in 2% (*w*/*v*) sodium dodecyl sulphate and 100 mm β-mercaptoethanol in 100 mM Tris–HCl (pH 7.0) for 60 min at 45 °C to remove adhering tissue residues. After extensive washing, the blots were reacted with affinity purified anti-GIRK2 antibodies (0.5 mg/mL) in blocking solution overnight at 4 °C. The bound primary antibodies were detected with alkaline phosphatase-conjugated anti-guinea pig IgG secondary antibodies [23]. To compare the expression levels of GIRK2 between (1) the wild type and P301S mice, and (2) wild type and APP/PS1 mice, at the experimental ages, all nitrocellulose membranes were processed in parallel, and the same incubation time for each reagent was used for the antibody. Digital images were acquired by scanning the nitrocellulose membranes using a desktop scanner (HP Scanjet 8300). Image analysis and processing were performed using the Adobe Photoshop software (Adobe Systems, San Jose, CA, USA) as described previously [27]. A series of primary and secondary antibody dilutions and incubation times were used to optimise the experimental conditions for the linear sensitivity range of all reactions and to confirm that all labelling was below saturation levels.

### 4.4. Immunohistochemistry for Electron Microscopy

Immunohistochemical reactions at the electron microscopic level were carried out using the pre-embedding immunogold as described earlier [56]. Briefly, free-floating sections obtained from wild type and P301S mice at 10 months, and from wild type and APP/PS1 mice at 12 months, were incubated in parallel in 10% (*v*/*v*) NGS diluted in TBS. Sections were then incubated in anti-GIRK2 antibodies (3–5 μg/mL diluted in TBS containing 1% (*v*/*v*) NGS), followed by incubation in goat anti-guinea pig IgG coupled to 1.4 nm gold (Nanoprobes Inc., Stony Brook, NY, USA). Sections were postfixed in 1% (*v*/*v*) glutaraldehyde and washed in double-distilled water, followed by silver enhancement of the gold particles with an HQ Silver kit (Nanoprobes Inc.). Sections were then treated with osmium tetraoxide (1% in 0.1 M phosphate buffer), block-stained with uranyl acetate, dehydrated in graded series of ethanol and flat-embedded on glass slides in Durcupan (Sigma-Aldrich, St. Louis, MO, USA) resin. Regions of interest were cut at 70–90 nm on an ultramicrotome (Reichert Ultracut E, Leica, Vienna, Austria) and collected on single slot pioloform-coated copper grids. Staining was performed on drops of 1% aqueous uranyl acetate followed by Reynolds’s lead citrate. Ultrastructural analyses were performed in a JEOL-1400Flash electron microscope (Jeol Ltd., Tokyo, Japan).

### 4.5. Quantification and Analysis of Pre-Embedding Immunogold Labelling

To establish the relative abundance of GIRK2 immunoreactivity in different compartments of CA1 pyramidal cells between wild type and P301S mice at 10 months, and from wild type and APP/PS1 mice at 12 months, we used 60-μm-thick coronal slices processed for pre-embedding immunogold immunohistochemistry. The procedure was similar to that as described previously [56]. Briefly, for each of three animals per experimental group, three samples of tissue were obtained for the preparation of embedding blocks. To minimise false negatives, electron microscopic serial ultrathin sections were cut close to the surface of each block, as immunoreactivity decreased with depth. We estimated the quality of GIRK2 immunolabelling by always selecting areas with optimal gold labelling at approximately the same distance from the cutting surface. Randomly selected areas were then photographed from the selected ultrathin sections and used with final magnification between 30,000 and 50,000 X. Quantification of immunogold labelling for GIRK2 was carried out in reference areas of the CA1 region totalling approximately 2000 µm^2^. We counted immunoparticles identified in each reference area and present in different subcellular compartments: dendritic spines, dendritic shafts, and axon terminals. The data were expressed as a percentage of immunoparticles for GIRK2 in each subcellular compartment, both in the plasma membrane and at intracellular sites.

### 4.6. Controls

To test method specificity in the procedures for histoblot and electron microscopy, the primary antibody was either omitted or replaced with 5% (*v*/*v*) normal serum of the species of the primary antibody, resulting in total loss of the signal. For the pre-embedding technique, labelling patterns were also compared with those obtained with Calbindin (polyclonal rabbit anti-Calbindin D-9k CB9; Swant, Marly, Switzerland); only the antibodies against GIRK2 consistently labelled the plasma membrane.

### 4.7. Data Analysis

To avoid observer bias, we performed blinded experiments for histoblots and immunohistochemistry prior to data analysis. Statistical analyses were performed using GraphPad Prism (San Diego, CA, USA) and data were presented as mean ± SEM unless indicated otherwise. Statistical significance was defined as *p* < 0.05. The statistical evaluation of the histoblots was performed using the Multiple *t*-test, with Holm-Sidak correction for multiple comparisons. To compute SEM error bars, three blots were measured from each animal.

## Data Availability

All data used and/or analysed during the current study are available from the corresponding author on reasonable request.

## Supplementary Materials

The following are available online at https://www.mdpi.com/article/10.3390/ijms222011106/s1.
