# Supplementary material for: The Expression and Localisation of G-Protein-Coupled Inwardly Rectifying Potassium (GIRK) Channels Is Differentially Altered in the Hippocampus of Two Mouse Models of Alzheimer’s Disease
Source: Int J Mol Sci. 2021 Oct 14;22(20):11106. doi: 10.3390/ijms222011106 (PMC8541655; doi:10.3390/ijms222011106)
Supplement: Supplementary file 1 [file ijms-22-11106-s001.zip › ijms-1413197-supplementary.pdf]

The expression and localisation of G-protein-coupled inwardly rectifying potassium (GIRK) channels is differentially altered in the hippocampus of two mouse models of Alzheimer´s disease

Rocío Alfaro-Ruiz et al.

SUPPLEMENTARY FIGURE S1

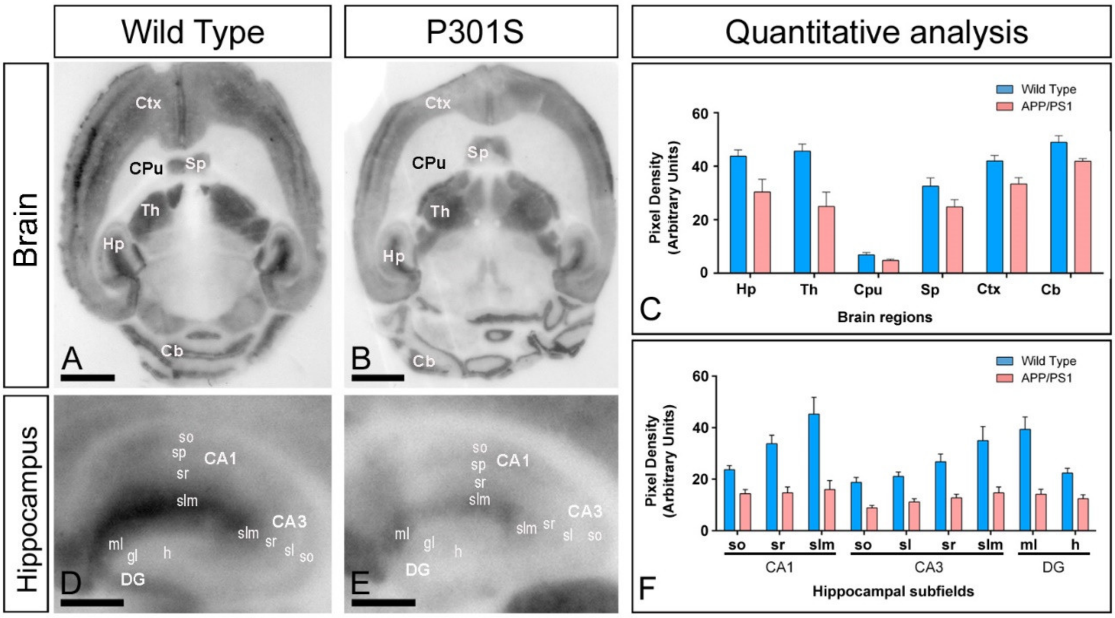

Supplementary Figure S1. ***Brain and hippocampal expression of GIRK1 in P301S mice***. (**A,B**) The expression of the GIRK1protein was visualised in histoblots of horizontal brain sections at 10 months of age in wild type and P301S mice using an affinity-purified anti-GIRK1 antibody. GIRK1 exhibited broad distributions in the brain. Strong GIRK1 staining was observed in the hippocampus (Hp), neocortex (Ctx), cerebellum (Cb), septum (Sp) and thalamus (Th), with moderate staining in midbrain nuclei and faint in the caudate putamen (CPu). (**D,F**) GIRK1 expression in the CA1 region was strong in the *stratum lacunosum–moleculare* (slm) and distal half of the stratum *radiatum* (sr) and moderate in the proximal half of the stratum *radiatum* and the *stratum oriens* (so). In the CA3 region, expression for GIRK2 was strong in the *strata lucidum* (sl), *radiatum* (sr) and *lacunosum–moleculare* (slm), and moderate in the *stratum oriens* (so). In the DG, GIRK2 expression was strong in the molecular layer (ml) and weak in the hilus (h). (**C,F**) Densitometric analysis at 10 months showed a significant decrease in the hippocampus and some of its layers (Multiple t-tests and Holm-Sidack method, * P<0,05). Error bars indicate SEM. *Abbreviations*: CA1 region of the hippocampus; CA3, CA3 region of the hippocampus; DG, dentate gyrus; so, *stratum oriens*; sp, *stratum pyramidale*; sr, *stratum radiatum*; slm, *stratum lacunosum-moleculare*; ml, molecular layer; gl, granule cell layer; h, hilus. Scale bars: 0.05 cm. Scale bars: A,B: 0.2 cm; D,F, 0.05 cm
